# Color-Vision and Color-Blindness

**Published:** 1896-01

**Authors:** 


COLOR-VISION AND COLOR-BLINDNESS. By Jennings. Published by The F. A. Davis Co., Philadelphia.

A practical manual for railroad surgeons. By J. Ellis Jennings, M. D. (University of Pennsylvania), formerly Clinical Assistant Royal London Ophthalmic Hospital (Moorfields); Lecturer on Ophthalmoscopy, and chief of the Eye Clinic in the Beaumont Hospital Medical College; Ophthalmic and Aural Surgeon to the St. Louis Mullanphy and Methodist Deaconness Hospitals; Consulting Oculist to the Missouri, Kansas, and Texas Railway System; Fellow of the British Laryngological and Rhinological Association; Secretary of the St. Louis Medical Society. This book is published as a convenient practical manual to aid the oculist, railroad surgeon, and general practitioner in accurately determining the fitness or unfitness of those employed, or seeking employment, in railway, steamboat, and steamship lines of transportation. In one crown octavo volume of over 100 pages. Illustrated with twenty-one (21) engravings and one (1) handsome full-page colored plate. Extra cloth, $1.00. Ready in January.

